# Cardiovascular Magnetic Resonance Elastography: Current Evidence, Challenges, and Future Perspectives

**DOI:** 10.3390/diagnostics16142233

**Published:** 2026-07-16

**Authors:** Alexander Wollandt, Leon D. Gruenewald, Christian Booz, Jennifer Gotta, Teodora Biciusca, Simon Bernatz, Philipp Reschke, Simon S. Martin, Tatjana Gruber-Rouh, Katrin Eichler, Iris Burck, Scherwin Mahmoudi, Mohamed Alrahmoun, Tommaso D’Angelo, Thomas J. Vogl, Sebastian Haberkorn, Marco Ochs, Hatim Kerniss, David M. Leistner, Omar Darwish, Radhouene Neji, Ralph Sinkus, Maria Johanna Gobertina Tetuanui Vehreschild, Ralph Strecker, Vitali Koch

**Affiliations:** 1 Clinic for Radiology and Nuclear Medicine, University Hospital, Goethe University Frankfurt, 60590 Frankfurt am Main, Germany; 2 Department of Cardiology, University Hospital Frankfurt, 60590 Frankfurt am Main, Germany; 3 School of Biomedical Engineering and Imaging Sciences, King’s College London, London SE1 7EH, UK; 4 Laboratory of Imaging Biomarkers, Center for Research on Inflammation, Unité Mixte de Recherche (UMR) 1149, Institut National de la Santé et de la Recherche Médicale (Inserm), Université de Paris Cité, 75018 Paris, France; 5 Department of Internal Medicine, Infectious Diseases, University Hospital, Goethe University Frankfurt, 60590 Frankfurt am Main, Germany; 6 EMEA Scientific Partnerships, Siemens Healthineers AG, 91052 Erlangen, Germany

**Keywords:** magnetic resonance elastography, cardiovascular disease, cardiac elastography, myocardial stiffness, aortic elasticity

## Abstract

Myocardial and aortic stiffness are increasingly recognized as clinically relevant biomarkers for cardiovascular disease, yet their noninvasive quantification remains challenging. Magnetic resonance elastography (MRE) has emerged as a promising technique for the spatial mapping of tissue biomechanics by visualizing and analyzing propagating shear waves. This narrative review summarizes the current state of cardiovascular MRE, spanning technical developments in wave generation (acoustic, electromagnetic, gravitational, and transducer-free approaches), pulse sequence design (echo-planar imaging, gradient-recalled echo, spiral, and free-breathing three-dimensional acquisitions), and inversion algorithms (local frequency estimation, direct inversion, finite element methods, and multifrequency elastography). We present evidence from phantom validation, animal models (myocardial infarction, hypertension, right ventricular hypertrophy), and human studies encompassing cardiac amyloidosis, hypertrophic cardiomyopathy, diastolic dysfunction, and abdominal aortic aneurysm. Additionally, we discuss current challenges, including waveguide effects, standardization needs, and clinical translation barriers, and highlight emerging solutions through artificial intelligence, multifrequency methods, and transducer-free cardiac MRE.

## 1. Introduction

Magnetic resonance elastography (MRE) has attracted scientific attention for its potential to address the unmet need for the detailed, noninvasive characterization of cardiac tissue biomechanics. While in the early stages of cardiovascular pathologies, no apparent symptoms may be present, the biomechanical properties of the myocardium and aorta are frequently altered in affected patients, often manifesting as elevated tissue stiffness and increased severity with further progression and longer duration of the underlying diseases [1,2,3]. Therefore, myocardial stiffness may not only play an important role in assessing cardiac function in ischemic cardiovascular diseases, hypertrophic cardiomyopathy (HCM), diastolic relaxation disorders, heart failure, and hypertensive heart disease, but also serve as a biomarker for detecting early tissue damage and monitoring disease progression [1,4,5,6,7,8,9,10].

MRE is already being used clinically to evaluate patients with chronic liver disease and is becoming a reliable noninvasive alternative to liver biopsy as a safe procedure for staging liver fibrosis, with many more studies underway to assess MRE in other organ pathologies, such as the brain, breast, fat tissue, kidney, lung, prostate, and skeletal muscle [4,11,12,13,14,15,16,17,18].

The current clinical assessment of myocardial stiffness relies primarily on invasive pressure-volume loop analysis via cardiac catheterization, which is limited by its invasive nature, procedural risks, and impracticality for serial monitoring [19]. Noninvasive surrogate markers derived from echocardiographic diastolic function parameters (*E/e*′ ratio, strain imaging) provide indirect estimates but do not directly measure intrinsic tissue mechanical properties. Late gadolinium enhancement (LGE) and T1 mapping on cardiac magnetic resonance imaging (MRI) can detect myocardial fibrosis, yet these approaches reflect tissue composition rather than biomechanical behavior [20,21,22,23,24]. Histological confirmation through endomyocardial biopsy, while considered the gold standard for fibrosis detection, is invasive, carries risks of bleeding and tissue damage, and may miss the focal pathology due to sampling errors [25].

Recent studies have demonstrated the feasibility of detecting both externally induced and intrinsic shear waves in the human heart using MRE, thereby enabling noninvasive quantitative assessment of myocardial stiffness [1,4,26,27,28,29]. Complementary approaches using ultrasound-based shear-wave elastography (SWE) have further demonstrated the clinical relevance of myocardial stiffness as a measurable biomarker, with studies reporting elevated shear-wave velocities in HCM, cardiac amyloidosis, and age-related stiffening [9,30,31].

This review article provides a comprehensive assessment of current evidence and technical developments in cardiovascular MRE, including wave-generation technology, pulse-sequence design, inversion algorithms, and clinical applications for cardiac and aortic tissue characterization. We discuss the current state of evidence from phantom, animal, and human studies; address existing technical and clinical challenges; and outline future perspectives for clinical translation, illustrated by three representative clinical cases that demonstrate the potential added value of MRE in diagnostic assessment and clinical decision-making.

## 2. Literature Search Methodology

Before presenting the technical foundations of MRE and its cardiovascular-specific features, we briefly outline the literature search strategy and review approach used in this narrative review.

Because of the broad methodological and thematic scope of MRE, as well as the heterogeneity of available study designs, no systematic review protocol (e.g., PRISMA or RPOSPERO) was applied. The literature search was performed primarily in PubMed while using a combination of the terms *magnetic resonance elastography, cardiovascular disease, cardiac elastography, myocardial stiffness, aortic elasticity, cardiovascular magnetic resonance elastography, aortic magnetic resonance elastography*, and *abdominal aortic aneurysm*. No predefined protocol was used while conducting the search, and combinations were adapted and updated throughout the writing process. The chosen studies were included based on their thematic relevance to cardiac MRE; experimental approaches to phantom, animal, and human studies; or technological developments in wave generation, sequence design, and inversion techniques. Only peer-reviewed literature was considered, placing a particular emphasis on screening recent studies given the rapid pace of development in MRE.

## 3. MRE

Essentially, MRE is an MRI technique that visualizes propagating mechanical waves within the organs under study, providing a quantitative assessment of tissue characteristics [32]. Central to this method are three components, namely wave generation, a specialized MRI sequence, and a calculation of tissue movement to visualize the data, all of which will be introduced and discussed in the following paragraphs. Applying mechanical shear waves to the tissue of interest generates small displacements known as shear waves. The continuous application of these waves enables measurement of their propagation speed, which is influenced by the stiffness of the medium [33]. To induce shear waves of varying frequencies in specific organs, an external transducer—or, more recently, endogenous structures such as the aortic valve are utilized [33,34,35,36,37]. The transducers are synchronized to magnetic resonance (MR) pulse sequences that employ motion-encoding gradients (MEG) and acquire wave propagation through the tissue at different time points. The resulting data are then processed using a mathematical inversion algorithm to generate quantitative images of tissue stiffness [1,11]. The inversion algorithm estimates the magnitude of the complex shear modulus, which reflects both the elastic and viscous properties of the tissue and is quantified in kPa [33].

The concept of MRE was first introduced by Muthupillai et al. in 1995, who demonstrated that propagating acoustic strain waves in tissue could be visualized using a phase-contrast MRI technique, enabling the noninvasive quantitative assessment of tissue mechanical properties [34]. Since then, MRE has evolved from a promising laboratory technique into the clinical tool it is today. Beyond proving itself to be a reliable means of diagnosing and staging liver fibrosis, it has garnered international interest, resulting in approval by regulatory bodies worldwide [38]. With an expanding portfolio of applications across organ systems and a rapidly growing number of publications each year, MRE has cemented its status as a highly relevant field of study [32].

Before further examination of the technical aspects of MRE, central mechanical terms need to be defined in accordance with the recently published International Society for Magnetic Resonance in Medicine (ISMRM) MRE Study Group consensus on principles, guidelines, and terminology [32]. Biological tissues are viscoelastic, meaning that they exhibit both elastic (energy-storing) and viscous (energy-dissipating) behavior during deformation.

The complex shear modulus G* = *G*′ + i*G*″ captures both properties, where the storage modulus *G*′ (elasticity) describes the ability of tissue to store mechanical energy and return to its original shape, and the loss modulus *G*″ (viscosity) reflects internal friction and damping during deformation, as well as the repartition of energy in space due to scattering [32,39]. “Stiffness” in the context of MRE typically refers to the magnitude of the complex shear modulus |G*|. In the absence of loss (*G*″ = 0), stiffness (often denoted as µ) is linked to the speed of the shear wave $c_{s}$ (“SWS”) through

(1) $$µ=\rho c_{s}^{2},$$

where *ρ* is the tissue density (conventionally assumed to be 1000 kg/m^3^) [32]. In practice, many cardiac MRE studies report shear-wave speed as the primary outcome, as it is experimentally measured and mathematically linked to shear stiffness, assuming negligible loss effects. Importantly, tissue mechanical properties are frequency-dependent (viscoelastic dispersion), meaning that stiffness changes with frequency [32,40]. This frequency dependence has particular implications for cardiac MRE, where different research groups have employed excitation frequencies ranging from 24 Hz to 220 Hz [1]. The Manduca et al. consensus recommends that all MRE studies report the excitation frequency alongside stiffness values to facilitate cross-study comparisons [32].

Beyond frequency dependency, variability in acquisition and reconstruction methodologies and reporting conventions further limits comparability among reported values. As a consequence, the reported stiffness values need to be interpreted as distinct physical quantities (stiffness in kPa or wave speed in m/s), whose relationships depend on the details of the dispersion properties. Universally accepted normative ranges have not yet emerged, highlighting the current lack of methodological standardization in cardiac MRE.

## 4. Wave Generation

**Figure 1:**
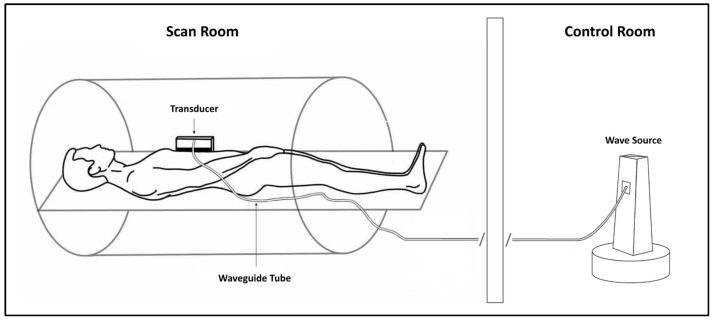
Example of an MRE transducer setup. This schematic illustration demonstrates the mechanical coupling between the vibration source and the imaging subject. A transducer is placed on the target area and fixed in place to ensure stable wave transmission and to prevent motion-related artifacts. In acoustic MRE systems, vibrations are generated by a remote actuator (“wave source”) located in close proximity. These vibrations are then transmitted via a flexible waveguide to the transducer, which conveys them to the tissue. In electromechanical MRE systems, the physical arrangement is very similar, even though vibrations are produced in the transducer. The transducer is again positioned on the target area and is connected to a motor by way of a flexible drive shaft, which transmits rotational movement to the transducer. Abbreviations: magnetic resonance elastography, “MRE”.

An MRE transducer system typically consists of a wave generator, an MR-compatible medium to deliver the generated shear waves to the target tissue, and finally a transducer positioned on the target area (Figure 1). Electromechanical (electromagnetic, piezoelectric, inertia/gravitational (GT)) and acoustic transducers are two of the most commonly used approaches for wave generation, although intrinsic cardiac motion-based MRE has also been explored. An acoustic transducer includes a drum-like passive transmission device to transfer waves generated by acoustic speaker systems and is widely used in MRE. The main advantage of acoustic transducers is their ease of handling and positioning, owing to their flexible design. Due to its flexibility and the good propagation of waves up to 140 Hz in the cardiovascular system, the acoustic transducer is frequently used in cardiovascular MRE studies [1,41,42,43,44,45]. Possible disadvantages of acoustic transducers include the risk of passive driver silencing due to preload (e.g., tight positioning or patient weight) and the potential imprecision of vibration waves associated with the inherent nonlinear properties of an acoustically driven system. Both problems may affect wave quality and hinder the accurate reconstruction of the final viscoelastic maps [46].

**Figure 2:**
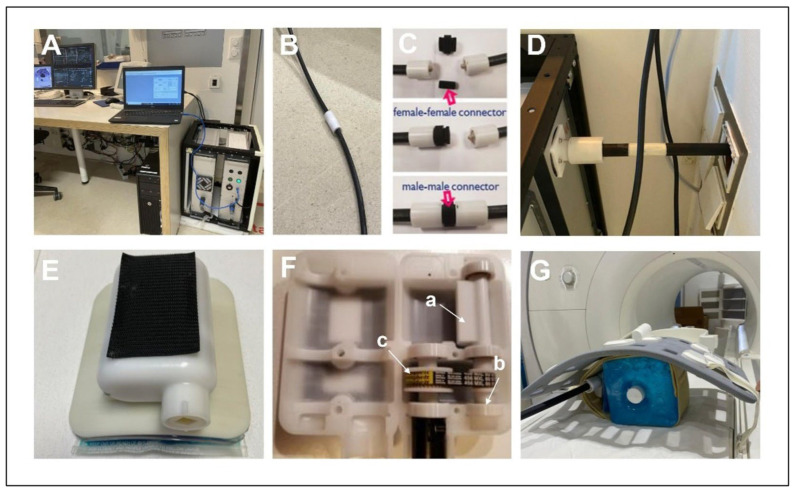
GT transducer setup. (**A**) The main board of the GT transducer, which includes a 4 nm stepper motor and a control unit, is located next to the main console of the MRI. This placement makes it convenient to program the system outside the MRI exam room and adjust the settings of the MRI sequences. (**B**,**C**) The system includes a flexible PEEK rotation axis. (**D**) The flexible waveguide is connected to the back-end motor and leads into the (**G**) MRI examination room to the transducer (**E**), which is positioned on a phantom in this example. (**F**) The transducer housing consists of two PEEK rods, one of which is bonded with (a) a polytetrafluoroethylene eccentric compound, and both rods are fitted with (b) two PEEK bearings. The two PEEK rods are linked together via (c) two PEEK timing pulleys and a timing belt. Abbreviations: gravitational, “GT”; magnetic resonance imaging, “MRI”; polyether ether ketone, “PEEK”.

Runge et al. have presented a new GT MRE transducer design based on the inertial forces of an eccentric rotating mass that generates highly accurate oscillatory waves without significant second-harmonic oscillations and can be precisely phase-locked to an MRE sequence (Figure 2) [46]. The GT concept merges the precision of electromechanical transducers with the artifact-free production of shear waves in acoustic devices. In addition, using a rotating eccentric mass results in a constant transducer vibration amplitude that is independent of the drive frequency, unlike other transducers, which tend to decrease in amplitude as the drive frequency increases [46]. As a result, the waves can be transmitted deep into the body or into organ parenchyma, even at higher frequencies, where a reduced amplitude is often a limitation for other transducers. Studies have demonstrated that the GT transducer achieves high accuracy, as evidenced by its strong suppression of upper harmonics relative to commercially available transducers [47]. It can also provide a constant amplitude across a wide range of frequencies used in clinical MRE applications (i.e., 40–70 Hz). The GT transducer concept was successfully tested for application on the liver in both phantoms and in vivo, with further clinical studies needed to assess the performance in cardiovascular imaging [46,48].

**Figure 3:**
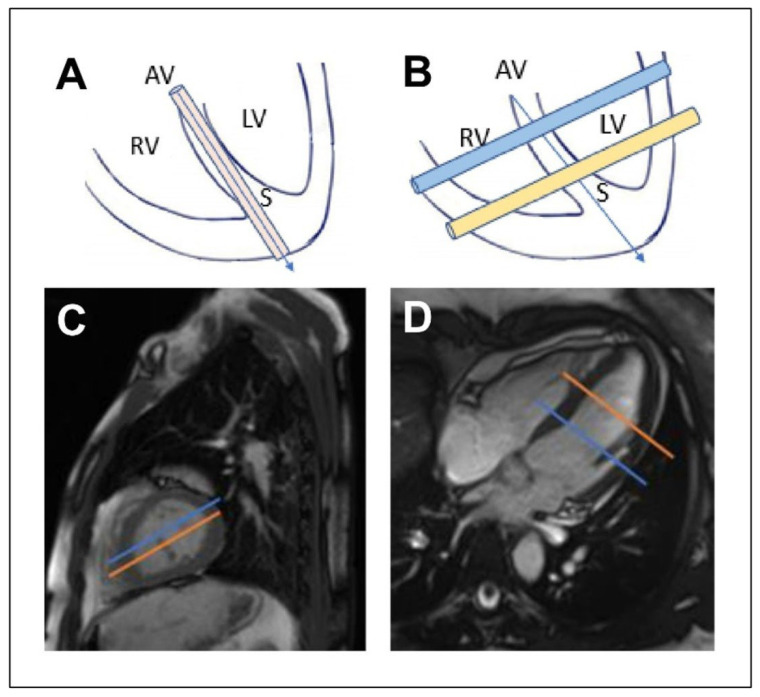
Example of in vivo positioning of the pencil beam. (**A**) At the moment of aortic valve closure, mechanical shear waves are generated and propagate from the base toward the apex of the heart (direction indicated by the blue arrow). Therefore, the 2D volume of the pencil beam (the bar overlapping the interventricular septum) was first projected within the interventricular septum positioned towards the apex base direction. (**B**) To mitigate partial volume effects and motion during image analysis, two pencil-beam volumes were positioned perpendicular to the interventricular septum, one at the base (blue bar) and one at the apex (orange bar). An in vivo positioning example is shown in a subject, depicting the short-axis view (**C**) and the horizontal long-axis view (**D**). Figure adapted from Ref. [4]. Abbreviations: two-dimensional, “2D”; aortic valve, “AV”; left ventricle, “LV”; right ventricle, “RV”; interventricular septum, “S”.

Whereas classic MRE uses an externally attached transducer on the target area, transducer-free approaches exploit intrinsic cardiac motion as the wave source, thereby circumventing many of the challenges associated with external transducers, including thoracic damping, frequency-dependent amplitude loss, and the need for additional hardware. Troelstra et al. demonstrated the feasibility of measuring the myocardial shear-wave velocity in vivo without an external transducer by utilizing the mechanical impulse of aortic valve closure as a wave source. Shear waves generated in this way propagate from the base to the apex at approximately 200–300 Hz, corresponding to the natural frequency content of valve closure [4]. Figure 3 illustrates this approach. The pencil beam was initially positioned within the interventricular septum in the apicobasal direction, but a perpendicular orientation to the interventricular septum—one at the base and one at the apical septum—resolved image analysis problems caused by partial volume effects and motion. This enabled a time-of-flight measurement of the shear-wave velocity between the pencil-beam volumes. As the spatial distance between the beams is precisely defined by the experimental setup, the travel time of the shear wave from one beam to the next can be measured with a temporal resolution of up to 0.3 ms. To compensate for the waveguide effect introduced by the geometric constraint whereby the wavelength exceeds the wall thickness, anatomical measurements from cine MRI enabled quantification of the wall thickness and cleared the measured wave speed of its geometrical bias. The corrected septal shear-wave velocity was significantly higher in patients with a cardiac pathology than in healthy volunteers (14.1 versus 3.6 m/s; *p* = 0.001) [4].

In parallel, ultrasound-based natural shear-wave imaging exploits both aortic and mitral valve closure to assess myocardial stiffness using high-frame-rate echocardiography (>1000 fps). Santos et al. established normal shear-wave velocity reference values (mitral valve closure: 3.2 ± 0.6 m/s; aortic valve closure: 3.5 ± 0.6 m/s) in 30 healthy volunteers with good reproducibility (intraobserver intraclass correlation coefficient (ICC) ~0.90) [31]. These complementary MR-based and ultrasound-based transducer-free approaches demonstrate the clinical relevance of intrinsic cardiac motion as a natural wave source for elastography.

For aortic MRE, Schaafs et al. employed a novel pressurized-air system with four 3D-printed passive actuators mounted beneath the subject, thereby providing multipoint excitation to improve aortic wave generation [49]. Anders et al. similarly used four compressed-air actuators positioned anterior and posterior to the heart and demonstrated that endogenous cardiac shear waves (without external vibration) are also suitable for cardiac stiffness mapping, albeit with lower reproducibility compared to externally driven approaches (ICC 0.72 vs. 0.93 for isovolumetric contraction) [50].

A comparative overview of wave generation methodologies is presented in Table 1, which outlines their advantages and disadvantages.

## 5. Cardiac MRE Sequences and Gradient Encoding

Under simple conditions, the square of the propagation speed yields the magnitude of the shear modulus of the tissue—hence, its stiffness [57,58]. The displacement field is extracted to estimate the shear modulus. An MR image acquired in this way, which contains information about the propagating wave in its phase, is called a wave image. Usually, two such wave images with opposite polarities for the MEG are obtained, and a phase-difference image is derived to eliminate non-motion-related phase information [1].

Cardiac MRE sequences are typically electrocardiogram (ECG)-gated and use either breath holds or navigators to avoid motion artifacts from respiration and the heartbeat. Additionally, because images spanning multiple stages of the cardiac cycle must be acquired alongside numerous phases of external motion, the sequences must be fast enough to capture all data in a single breath hold [1].

To enable fast imaging and minimize sensitivity to cardiac motion, a commonly employed pulse sequence for cardiac MRE is single-shot spin-echo echo-planar imaging (SS-SE-EPI) [59].

**Figure 4:**
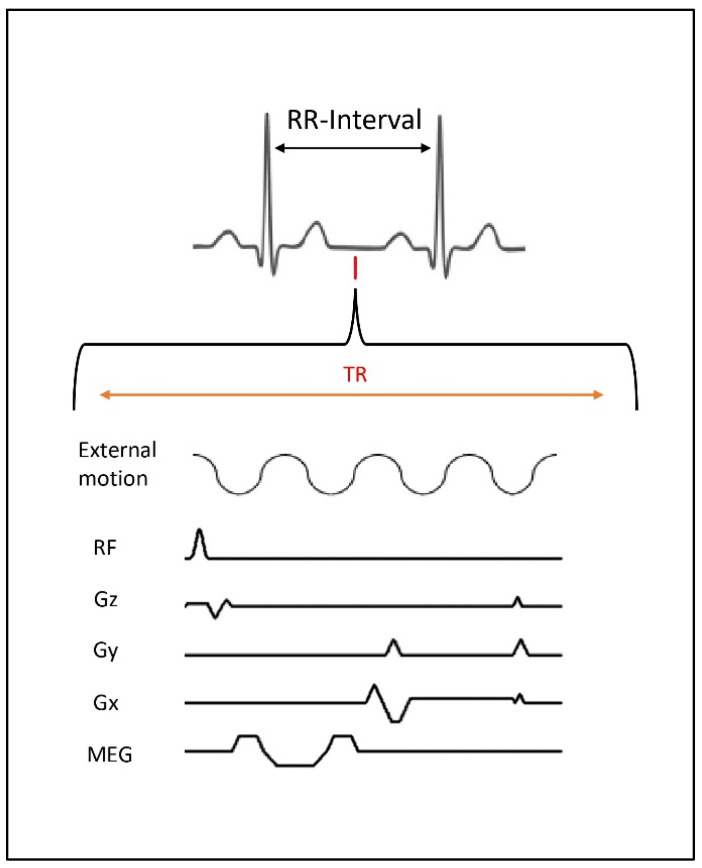
Schematic illustration of a GRE pulse sequence used for cardiac MRE. The diagram shows the temporal relationship between cardiac motion (RR-interval) and the sequence components (red indicator below the ECG-curve). Continuous mechanical excitation is applied throughout the cardiac cycle, while a radiofrequency (RF) excitation pulse initiates signal generation. Imaging gradients, Gx, Gy, and Gz, encode unique information for each of their corresponding geometrical directions. The first-moment nulled MEG, which can be applied on any axis, is applied after the RF excitation of the sample and before the measurement of the induced signal. The fractionally encoded signal is assessed eight times per mechanical vibration, and the process is repeated throughout the cardiac cycle. TR represents the repetition time between RF excitations in a GRE sequence [1,60]. Figure created based on data from Ref. [60]. Abbreviations: electrocardiogram, “ECG”; imaging gradient in x-direction, “Gx”; imaging gradient in y-direction, “Gy”; imaging gradient in z-direction, “Gz”; gradient-recalled echo, “GRE”; motion encoding gradient, “MEG”; magnetic resonance elastography, “MRE”; radiofrequency, “RF”; interval between two subsequent R-waves, “RR-Interval”; time of repetition, “TR”.

Alternatively, gradient-recalled echo (GRE) sequences offer shorter echo times and greater flexibility in acquisition timing across the cardiac cycle and have also been widely adopted for cardiac MRE. Kolipaka et al. developed a retrospectively gated multiphase GRE MRE sequence for cardiovascular imaging (Figure 4) [1,60]. A recent study by Meyer et al. showed the feasibility of free-breathing 3D MRE stiffness mapping of the left ventricular (LV) myocardium in 11 healthy volunteers at 100 Hz. By using a 3D hybrid radial and echo-planar imaging (EPI) acquisition protocol (TURBINE_EPI) with retrospective physiologic binning into seven cardiac phases, this approach eliminates the need for breath-holds entirely. The mean stiffness ranged from 3.5 kPa (mid-diastole) to 5.7 kPa (mid-systole), with results comparable to those of a breath-hold spin-echo echo-planar imaging (SE-EPI) reference [52].

Beyond EPI-based readouts, spiral k-space trajectories have recently been applied to cardiac MRE. Anders et al. developed a 2D multifrequency spiral cardiac MRE sequence (70/80/90 Hz), achieving a 40 Hz temporal resolution with a 2.0 mm in-plane resolution, enabling time-resolved myocardial stiffness mapping across the cardiac cycle in a single breath hold of approximately 23 s [50]. This approach demonstrated excellent reproducibility during the isovolumetric contraction phase (ICC = 0.93) and revealed that SWS peaks at end-systole (2.15 ± 0.23 m/s) and is lowest during isovolumetric contraction (1.76 ± 0.17 m/s) [50].

Sui et al. further advanced cardiac MRE acquisition by introducing a reduced field of view (rFOV) approach using 2D spatially selective radiofrequency excitation, which significantly reduced Nyquist ghosting artifacts and improved the signal quality (OSS-SNR 2.1 vs. 1.4 for full field of view (FOV)) (*p*  <  0.05) without affecting the measured stiffness values [59].

In their transducer-free approach to shear-wave velocity measurements, which relies on intrinsic cardiac wave generation, Troelstra et al. employed an ECG-gated sequence during a breath hold of approximately 15 cardiac cycles, using a linear 2D pencil-beam excitation pulse. While this method provides a fast, highly reproducible means of sampling intrinsic cardiac shear waves, key elastographic parameters, such as the spatial resolution and viscoelastic assessment, remain inaccessible [4]. Table 2 compares the pulse sequences used in cardiac MRE studies to date.

Collectively, these sequence developments illustrate a trade-off between spatial resolution, temporal resolution, and acquisition speed, with no single approach yet established as a reference standard; direct head-to-head comparisons across sequences in the same cohort are lacking, limiting conclusions about which technique is optimal for clinical use.

Beyond cardiac applications, MRE sequences have also been adapted for assessing aortic stiffness. Schaafs et al. employed a pulse-triggered steady-state GRE MRE sequence with segmented spiral k-space readout (stroboscopic MRE) at three frequencies (50, 62.5, 80 Hz), enabling multifrequency aortic stiffness mapping with excellent inter- and intraobserver reproducibility (ICC 0.981–0.991) [49].

## 6. Elastographic Inversion Methods

After generating continuous harmonic motion in the tissue and encoding the resulting displacements with MEG, the elastic properties of the tissue must be derived from the acquired phase data via mathematical inversion [11,34,37]. Under the assumption of an isotropic, linearly elastic, and locally homogeneous viscoelastic material, the equation for harmonic motion in the frequency domain can be written as

(2) $$-\rho \omega^{2}\vec{U}=(\mu +\lambda )\nabla (\nabla \cdot \vec{U})+\mu \nabla^{2}\vec{U},$$

where μ and λ are the Lamé parameters. The shear modulus equals μ, whereas λ is the first Lamé parameter associated with volumetric (compressional) deformation, $\vec{U}$ ($\vec{r}$, *f*) represents the vector displacement at position $\vec{r}$, *ρ* is the tissue density (typically approximated at 1000 kg/m^3^), and

(3) ω=2πf

is the angular frequency of the applied harmonic motion [64]. Under the assumption of incompressibility (∇·$\vec{U}$ = 0) and a finite *λ*, Equation (2) simplifies to the vector Helmholtz equation, where *G* is the shear modulus [14,64]:

(4) $$-\rho \omega^{2}\vec{U}=G\nabla^{2}\vec{U}$$

Several inversion strategies have been developed to solve for the shear modulus *G* from measured displacement fields, each with distinct strengths and limitations for cardiovascular applications.

### 6.1. Direct Inversion (Algebraic Inversion)

The most straightforward approach is the direct algebraic inversion of the Helmholtz equation, where the shear modulus is calculated voxel-wise as

(5) $$G=\frac{-\rho \omega^{2}U_{n}}{\nabla^{2}U_{n}},$$

where n denotes a single component of the displacement vector. This method requires computing the Laplacian of each component of the displacement field, which involves second-order spatial derivatives and is therefore sensitive to noise [11]. Direct inversion has been applied in cardiac MRE by Arani et al. using 3D displacement fields acquired at 140 Hz, demonstrating accurate stiffness recovery in a diastolic cardiac phantom and feasibility in healthy volunteers [42].

### 6.2. Local Frequency Estimation (LFE)

LFE is a spatial frequency-based approach that estimates the local wavelength of the propagating shear wave from the displacement field using octave-band spatial filters [11]. The shear modulus is then derived from the wavelength and excitation frequency. LFE has been the most widely used inversion method in cardiovascular MRE, employed by Kolipaka et al. for both cardiac and aortic applications [26,41,61,65], by Wassenaar et al. for age-dependent myocardial stiffness mapping [29], in myocardial infarction (MI) and hypertensive models [27,28,62], and most recently by Meyer et al. for free-breathing 3D cardiac MRE [52]. LFE is relatively robust to noise compared to direct inversion, but it provides lower spatial resolution due to the filtering operation [11].

### 6.3. Spherical Shell Analysis

Kolipaka et al. introduced a specialized analytical inversion approach for cardiac MRE based on the equations of motion for a thin spherical shell, accounting for the curved geometry of the LV [26]. This approach uses in-plane radial and circumferential displacement components and has been validated against pressure–volume measurements, demonstrating excellent concordance (*R*^2^ = 0.97–0.98) under both static and cyclically varying pressure conditions [26]. The spherical shell method was subsequently used to demonstrate that the MRE-derived effective myocardial stiffness correlates linearly with the invasively measured LV pressure throughout the cardiac cycle (*R*^2^ = 0.84) [61].

### 6.4. Finite Element Method-Based Inversion

Finite element-based approaches solve the inverse elasticity problem by iteratively minimizing the difference between measured and simulated displacement fields within a discretized tissue model. Finite element method inversion can incorporate complex boundary conditions and realistic tissue geometries, thereby making it well-suited to thin-walled cardiac chambers in theory. Fovargue et al. developed a localized divergence-free finite element reconstruction that has been applied to cardiac MRE, demonstrating improved quantification of stiffness in the presence of boundary effects [47].

### 6.5. Tomoelastography (TMRE) (Multifrequency Wavenumber Inversion)

A recent advancement in cardiac MRE inversion is the TMRE approach, which utilizes a k-space-based multidirectional elasto-viscoelasticity reconstruction (k-MDEV) method. In TMRE, wave fields acquired at multiple frequencies are processed to extract local wavenumbers. These wavenumbers are then combined using amplitude-weighted averaging to produce high-resolution stiffness maps. Castelein et al. demonstrated that cardiac TMRE provided highly reproducible measurements of LV stiffness (ICC = 0.96) and viscosity (ICC = 0.93) in a same-day test–retest study using multifrequency excitation at 80, 90, and 100 Hz [63]. Anders et al. further showed that multifrequency spiral cardiac MRE with k-MDEV processing achieves a high spatiotemporal resolution (40 Hz frame rate) for time-resolved myocardial stiffness mapping across the cardiac cycle [50].

### 6.6. Waveguide Considerations in Cardiac MRE

A critical challenge for all cardiac MRE inversion methods is the waveguide effect arising from the thin-walled geometry of the heart. When the myocardial wall thickness is comparable to or smaller than the shear wavelength, wave propagation is governed by guided-wave (Lamb-wave) physics rather than bulk shear-wave propagation, leading to a systematic bias in conventional inversion algorithms. Manduca et al. demonstrated, through finite element simulations, that applying the curl operator to the displacement field and performing full 3D inversion are both necessary for accurate shear modulus estimation in cardiac MRE; 2D inversions, even with curl preprocessing, are insufficient for realistic cardiac geometries [66]. This finding has important implications for interpreting cardiac MRE stiffness values, as many early studies used 2D inversion methods. The curl operation removes the longitudinal (compressional) wave component, which would otherwise corrupt stiffness estimates through the dilatational term in the wave equation [1,32]. Troelstra et al. addressed the waveguide problem in their transducer-free approach by applying a geometry-based power-law correction to flexural-wave dispersion, thereby enabling more accurate conversion of the measured wave speed to tissue stiffness [4].

## 7. State of Evidence: Cardiac MRE

Cardiovascular elastography has proven difficult due to the relatively high stiffness, the thinness of the ventricular and septal walls, and the need to compensate for physiologic cardiac and respiratory motion during the cardiac cycle [4]. Therefore, few studies have been published on human cardiac elastography [1,13,44,53,67]. The implementation of cardiovascular MRE to measure myocardial elastic properties has remarkable potential to enable the noninvasive assessment of myocardial dysfunction and relaxation pathologies associated with increased myocardial stiffness [1].

One elastic property that shows a promising correlation with cardiovascular disease is myocardial SWS. Published data show that the velocity of myocardial shear waves is significantly higher in patients with cardiovascular disease than in healthy subjects (14.1 m/s versus 3.6 m/s, *p*  =  0.001) [4]. Although the shear-wave velocity is expected to be higher in patients with underlying heart disease, it is also possible that the myocardium takes longer to fully relax in these patients, implying that a portion of the increased shear-wave velocity may be ascribed to delayed relaxation. Another possible reason for the observed higher wave velocity may be the measurement timing within the cardiac cycle. In this context, myocardial stiffness changes depend on the ventricle’s filling and contraction during the cardiac cycle. The aortic valve closes at the end of systole and remains closed until the beginning of diastole. This means that the ventricle is still partly contracted, resulting in a higher wave velocity than in studies performed in diastole [4].

Due to the heart’s complex geometry, 3D displacement encoding and processing are essential for accurate stiffness calculations, as demonstrated by Manduca et al. in their finite element analysis of waveguide effects [42,66]. Frequency selection remains a critical consideration: lower vibrational frequencies penetrate more easily but yield longer wavelengths with a reduced spatial resolution and noise tolerance, while higher frequencies provide a better resolution at the cost of increased attenuation. Published studies have employed frequencies ranging from 24 Hz to 220 Hz for cardiac applications, with 80–140 Hz emerging as the most common range for external transducer approaches [1,42].

Research has progressed along two complementary paths, leading to the current understanding of the cardiac MRE landscape. Phantom and animal studies provide controlled environments and remain the foundation for novel approaches, while subsequent human studies assess feasibility and in vivo implementation. The following sections describe the status of development in both fields.

## 8. Phantom and Animal Studies

### 8.1. Phantom Validation

Phantom studies have played a critical role in validating cardiac MRE methodologies. Kolipaka et al. performed the seminal phantom validation using a silicone rubber sphere mimicking the LV geometry, demonstrating excellent concordance between MRE-derived stiffness and pressure–volume measurements under both static and cyclically varying pressure conditions (*R*^2^ = 0.97–0.98), establishing that cardiac MRE can estimate myocardial stiffness in a geometrically complex, fluid-filled, moving structure [26]. Arani et al. subsequently demonstrated accurate quantitative cardiac MRE stiffness maps in an anatomically precise diastolic cardiac phantom, showing that the accuracy improves with increasing excitation frequencies, with errors decreasing from approximately 50% at 80 Hz to less than 1% at 220 Hz, and identifying 140 Hz as the optimal frequency for in vivo application [42].

### 8.2. Animal Studies: Pressure–Stiffness Validation

Several animal studies have validated cardiac MRE against the invasive gold standard of pressure–volume loops. Kolipaka et al. demonstrated, in six pigs, that the MRE-derived effective myocardial stiffness varies cyclically throughout the cardiac cycle (end-systolic: 9.34 ± 1.9 kPa; end-diastolic: 6.03 ± 1.8 kPa), with a good linear correlation with the invasively measured LV pressure (*R*^2^ = 0.84), and that stiffness–volume loops show excellent visual agreement with pressure–volume loops [61]. In a follow-up study, they showed that the end-diastolic stiffness measured by MRE increased linearly with LV pressure across different loading conditions (*R*^2^ = 0.73–0.90 per animal), confirming its sensitivity to changes in passive myocardial stiffness [68]. They further demonstrated that the end-systolic MRE-derived stiffness increased with epinephrine infusion, independent of loading conditions, suggesting its utility as a noninvasive surrogate for myocardial contractility [69].

### 8.3. Disease Models

MI has been the most extensively studied disease model. Mazumder et al. demonstrated a progressive increase in infarct zone stiffness at 10 and 21 days post-MI in a porcine model (baseline diastolic 3.87 ± 0.4 kPa vs. day 21 infarct 5.45 ± 0.7 kPa; *p* < 0.05), while the remote myocardial stiffness remained unchanged. The MRE-derived stiffness correlated strongly with ex vivo mechanical testing (*r* = 0.86–0.89) and the extracellular volume fraction (*r* = 0.55–0.70), confirming fibrosis as the underlying mechanism [27]. Arunachalam et al. corroborated these findings using 3D MRE at 140 Hz with isotropic voxels, demonstrating significantly greater stiffness in the infarcted myocardium (4.6 ± 0.7 kPa) than in remote tissue (3.0 ± 0.6 kPa; *p* = 0.02) [28].

In a hypertensive porcine model, Mazumder et al. showed that MRE-derived myocardial stiffness increased significantly with the progression of hypertension over two months (end-diastolic: baseline 3.84 ± 0.4 to 4.82 ± 0.2 kPa; *p* < 0.0001), and it correlated positively with LV pressure and wall thickness [62]. da Silveira et al. demonstrated the initial feasibility of cardiac MRE for right-ventricular stiffness quantification in dogs with congenital pulmonary valve stenosis, reporting significantly higher free-wall stiffness in the right ventricle (RV) than the LV, with the RV stiffness correlating with the extracellular volume fraction (*r* = 0.75) (*p* = 0.05) [70].

### 8.4. Small-Animal Models

Liu et al. achieved the first cardiac MRE in a mouse model at 9.4 T using 400 Hz excitation, demonstrating cardiac cycle-dependent stiffness variations consistent with those in larger animals and establishing a platform for investigating cardiac disease in genetically engineered mouse models [71].

## 9. Human Studies

### 9.1. Healthy Volunteers and Normal Reference Values

Establishing normative values for myocardial stiffness is essential for clinical translation. Since the first human cardiac MRE feasibility demonstration by Elgeti et al. in 2008 (eight healthy volunteers, 24.3 Hz) [53], subsequent studies have reported LV stiffness values ranging from approximately 3.5 to 7.4 kPa across a range of actuation frequencies (80–220 Hz) and acquisition approaches, including standard 3D cardiac MRE [42,59], free-breathing TURBINE-EPI MRE [52], and acoustic transducer-based protocols [29]. The reported reproducibility has generally been good where assessed (concordance coefficients 0.77–0.93), and Wassenaar et al. additionally identified an age-related increase in the end-systolic–end-diastolic stiffness difference (0.014 kPa/year) using cardiac MRE at 80 Hz, reporting end-systolic stiffness of 6.10 ± 1.38 kPa and end-diastolic stiffness of 4.99 ± 1.05 kPa (*p* < 0.0001), with good intra- and intersession reproducibility (concordance coefficient 0.77 and 0.93, respectively) [29].

However, the considerable spread in absolute stiffness values across studies (e.g., 3.8 kPa at 140 Hz [42] vs. 7.2–7.4 kPa at the same frequency [59]) likely reflects differences in vibration hardware, sequence design, and analysis pipeline rather than true biological variability, underscoring the current absence of a standardized cardiac MRE acquisition and processing protocol—a prerequisite for establishing generalizable reference ranges.

In the largest cardiac MRE cohort study to date, Arani et al. measured the LV myocardial stiffness in 109 healthy volunteers (57 women, 52 men; age 18–84 years) using a 5 min MRE acquisition protocol at 140 Hz, added to a clinical MRI protocol [72]. Notably, myocardial stiffness significantly increased with age in female (slope 0.03 kPa/year, *p* = 0.009) but not male (slope 0.008 kPa/year, *p* = 0.38) volunteers, revealing a sex-specific pattern of age-related myocardial stiffening that was not detectable by conventional MRI parameters, including T1 mapping, strain, or LV mass [72]. This finding has important clinical implications, as the higher prevalence of heart failure with a preserved ejection fraction (HFpEF) in women may be mechanistically linked to this sex-differential stiffening pattern.

Notably, the multifrequency approach has shown promising reproducibility (ICC 0.93–0.96) in small volunteer cohorts (*n* = 11–18) [50,63], but, as with single-frequency protocols, cross-study comparability is limited by the lack of a harmonized acquisition standard, and validation in larger, more heterogeneous populations is still needed.

### 9.2. Cardiac Amyloidosis

Cardiac MRE has shown particular promise in cardiac amyloidosis, in which diffuse myocardial infiltration increases tissue stiffness. Arani et al. performed 3D cardiac MRE at 140 Hz in 22 patients with biopsy-proven cardiac amyloidosis (light-chain and transthyretin (TTR) subtypes) and 16 healthy controls, demonstrating significantly elevated median myocardial stiffness in patients (11.4 kPa, range 9.2–15.7) compared to controls (8.2 kPa, range 7.2–11.8; *p* = 0.0008) [51]. Chang et al. corroborated these findings in a feasibility study comparing a patient with hereditary TTR amyloidosis (15.7 kPa) with a healthy control (7.2 kPa) at 140 Hz [73].

Using ultrasound-based natural shear-wave imaging, Petrescu et al. reported significantly higher myocardial shear-wave velocities in 17 cardiac amyloidosis patients compared to 46 healthy volunteers (6.33 ± 1.63 vs. 3.54 ± 0.93 m/s after mitral valve closure; *p* < 0.001), with the velocities correlating with the diastolic dysfunction grade and *E/e*′ ratio (*r* = 0.74) [30]. Santos et al. similarly demonstrated markedly elevated natural shear-wave velocities in a patient with cardiac amyloidosis (6.6 m/s) compared with the healthy reference range (3.2 ± 0.6 m/s) [31]. Collectively, these findings are based on small, largely single-center cohorts (≤22 patients per study), and the reported stiffness cut-offs should be regarded as exploratory rather than validated diagnostic thresholds.

### 9.3. Hypertrophic Cardiomyopathy

Zhao et al. recently reported the first dedicated cardiac MRE study in HCM, demonstrating markedly elevated LV myocardial stiffness (21.8 kPa) compared to the published reference range (7.2–9.8 kPa) [74]. Using ultrasound-based acoustic radiation force impulse shear-wave imaging in a larger cohort, Villemain et al. measured myocardial stiffness in 20 patients with HCM HFpEF and 60 healthy volunteers, reporting substantially elevated stiffness in patients (12.68 ± 2.91 kPa) compared to controls (4.47 ± 1.68 kPa), with a cut-off of 8 kPa achieving near-perfect diagnostic accuracy (AUC = 0.993) [9]. Stiffness increased linearly with age in healthy adults (*R*^2^ = 0.77) and correlated strongly with echocardiographic diastolic parameters (*E/e*′, *r* = 0.783) and cardiac MR fibrosis markers (LGE, *r* = 0.804; native T1, *r* = 0.711) [9]. Burnhope et al. assessed the myocardial shear-wave velocity using the transducer-free approach in patients with HCM (15.2 ± 10.6 m/s; *p* < 0.001), hypertensive heart disease (7.7 ± 4.8 m/s; *p* < 0.004), and cardiac amyloidosis (14.0 ± 4.8 m/s; *p* < 0.001), demonstrating significantly increased velocities in all pathological groups compared to healthy controls [55]. As with the amyloidosis literature, these HCM findings, including the reported diagnostic cut-off of 8 kPa, are derived from single-center studies with limited patient numbers and require confirmation in larger, prospective cohorts before clinical application.

### 9.4. Diastolic Dysfunction and Heart Failure

Elgeti et al. investigated cardiac MRE at 24.13 Hz in 10 patients with mild diastolic dysfunction (predominantly hypertension-related) and 15 healthy volunteers, finding significantly reduced LV shear-wave amplitudes in patients (amplitude ratio 0.33 ± 0.08 vs. 0.62 ± 0.15 in young participants and 0.50 ± 0.09 in older participants; *p* < 0.001) [54]. In a subsequent study using amplitude-based cardiac MRE at 24.13 Hz, the shear-wave amplitudes were found to be significantly lower in patients with mild to severe diastolic dysfunction (mild 0.37 ± 0.04, moderate 0.34 ± 0.04, and severe 0.29 ± 0.04; *p* < 0.001) compared to healthy subjects, providing further evidence that cardiac MRE can detect early myocardial stiffness changes [67,75]. Meyer et al. recently demonstrated that point-of-care cardiac ultrasound elastography with external vibration can quantify diastolic myocardial stiffness, distinguishing wild-type transthyretin (wTTR) amyloidosis from healthy controls (SWS 3.0 ± 0.7 vs. 1.8 ± 0.3 m/s; AUC = 0.991) [76].

### 9.5. Emerging Applications and Clinical Translation

The transducer-free cardiac MRE approach introduced by Troelstra et al. represents a significant step toward clinical integration by eliminating the need for external hardware. Using aortic valve closure-induced shear waves, corrected septal shear-wave velocities were significantly higher in four patients with confirmed cardiac pathologies than in 12 healthy volunteers (14.1 vs. 3.6 m/s; *p* = 0.001) [4]. Castelein et al. recently argued that multiplex (multifrequency) approaches are essential for the clinical translation of cardiac MRE, citing the need for vendor-agnostic integrated vibration hardware and standardized processing pipelines [77].

To summarize, while disease-specific stiffness differences (e.g., in amyloidosis and HCM) have been shown to be reproducible across independent groups, the proposed diagnostic thresholds have not been prospectively validated in larger, multicenter, or histologically confirmed populations and should therefore be considered exploratory. Supplementary Table S1 provides a comprehensive overview of published human cardiac MRE and SWE studies.

## 10. State of Evidence: Aortic MRE

The application of MRE to the aorta presents unique challenges that are distinct from those in cardiac MRE. The thin aortic wall and surrounding blood require the exploitation of the waveguide effect, whereby the aortic wall and luminal blood vibrate at the same frequency, enabling stiffness maps to be generated from wave analysis within the entire aortic cross-section [1,78,79,80]. Aortic stiffness is a recognized cardiovascular risk marker, and gaining quantitative insight into regional aortic mechanical properties has potential value in differentiating and monitoring pathological changes in diseases such as Marfan syndrome, aortic aneurysms, atherosclerosis, and hypertension [81,82,83].

A reference table of representative aortic MRE studies is compiled in Supplementary Table S2, listing their study parameters and findings.

### 10.1. Phantom and Ex Vivo Validation

Zhang et al. established foundational evidence for regional aortic MRE using vascular phantoms with silicone tubes of varying stiffness embedded in gel, confirming that MRE can detect regional stiffness variations via both LFE and phase-gradient inversion at 60 Hz [78]. They further validated these findings in ex vivo porcine aortas, demonstrating that formalin-treated (stiffened) aortic segments showed significantly higher MRE-derived stiffness (19.3 ± 1.2 kPa; *p* = 0.05) compared to fresh tissue (8.8 ± 1.8 kPa), with the stiffness values correlating with mechanical testing (ρ = 0.73) [78]. Woodrum et al. demonstrated, in ex vivo hypertensive porcine aortas, that the MRE-measured stiffness was significantly higher in hypertensive specimens than in controls (0.571 ± 0.080 vs. 0.419 ± 0.026, *p* < 0.05), with histopathology confirming an increased intima–media thickness and greater collagen content [84].

### 10.2. Healthy Volunteers and Aging

Damughatla et al. performed aortic MRE and MRI-based pulse-wave velocity measurements in 21 healthy volunteers aged 18 to 65, reporting that both the MRE-derived stiffness and pulse-wave velocity increased linearly with age (*R*^2^ = 0.81 and 0.67, respectively). Young volunteers showed significantly lower aortic stiffness than older volunteers (4.5 ± 0.6 vs. 7.98 ± 1.6 kPa; *p* = 0.0004), although the MRE-derived and pulse-wave velocity-derived stiffness showed only a moderate correlation (*R*^2^ = 0.43), attributed to the different measurement frequencies (60 Hz vs. ~1 Hz cardiac cycle) [85]. Kenyhercz et al. further demonstrated periodic variations in aortic stiffness during the cardiac cycle in 20 healthy volunteers, with significantly higher values during end-systole than end-diastole (*p* < 0.0001) [86].

Kolipaka et al. conducted the first in vivo aortic MRE comparison between hypertensive and normotensive subjects, reporting significantly greater abdominal aortic stiffness in four controlled hypertensives than in four normotensives (9.3 ± 1.9 vs. 3.7 ± 0.8 kPa; *p* = 0.02) at 60 Hz [41]. Schaafs et al. subsequently applied steady-state multifrequency MRE to the thoracic and abdominal aorta in healthy volunteers, providing reference stiffness values across multiple frequencies and demonstrating the feasibility of TMRE processing for aortic applications [49].

### 10.3. Abdominal Aortic Aneurysm

Aortic MRE has shown particular promise for assessing abdominal aortic aneurysm (AAA), where biomechanical properties may provide superior rupture risk prediction compared with diameter alone. Kolipaka et al. performed in vivo aortic MRE in 24 AAA patients and 12 healthy volunteers at 60 Hz, finding significantly elevated AAA stiffness (13.97 ± 4.2 kPa; *p* ≤ 0.02) compared to remote normal aortas (8.87 ± 2.2 kPa) and healthy volunteers (7.1 ± 1.9 kPa). Importantly, the AAA stiffness did not correlate with the aneurysm diameter, suggesting that MRE may capture biomechanical information independently of anatomical measurements [65].

Dong et al. advanced aortic MRE through a series of studies progressing from animal validation to clinical outcomes. In a porcine AAA model, they demonstrated that MRE-derived stiffness increased by 41–66% in aneurysmal tissue compared to baseline, with stiffness correlating inversely with elastin density (ρ = −0.68, *p* < 0.0001) and positively with mineralization [43]. They subsequently validated cardiac-gated SE-EPI aortic MRE in 40 healthy subjects, confirming its excellent reproducibility (concordance coefficient 0.96–0.99) across free-breathing and single-breath-hold protocols [87]. In the most clinically significant aortic MRE study to date, Dong et al. performed aortic MRE in 72 AAA patients with longitudinal follow-up (~15 months), demonstrating that lower AAA stiffness and stiffness ratios were associated with aneurysmal events (rupture, repair, or diameter >5 cm), while stiffness did not correlate with the AAA diameter [88]. This finding supports the hypothesis that biomechanical assessment through MRE could complement diameter-based surveillance in AAA management.

At the microscopic level, Mangarova et al. employed multifrequency micro-MRE at 7 Tesla (1000–1400 Hz) to map the ex vivo AAA stiffness at a 40 µm resolution in a mouse model, demonstrating that SWS correlated strongly with the histologically quantified extracellular matrix (ECM) content (*R*^2^ = 0.79 for elastin and collagen), with ECM-rich thrombus regions showing significantly higher stiffness than ECM-poor regions (1.04 vs. 0.44 m/s; *p* < 0.05) [89].

## 11. Summary of Challenges and Outlook on Future Perspectives

### 11.1. Technical Challenges

Several fundamental technical challenges must be addressed for cardiovascular MRE to achieve widespread clinical adoption. The complex geometry of the heart, with its thin walls (typically 8–12 mm in diastole) and continuous thickness changes during the cardiac cycle, results in guided-wave (Lamb-wave) propagation rather than bulk shear-wave behavior at the frequencies currently employed (24–220 Hz) [1,66]. This waveguide effect introduces a systematic bias in conventional inversion algorithms that assume infinite, homogeneous media. While the curl operation and 3D inversion have been shown to mitigate these effects [66], these mathematical operations require highly resolved wave fields and are limited by the signal-to-noise ratio. Thus, the further development of geometry-aware inversions that incorporate the cardiac wall thickness and curvature is needed [1].

Transducer technology remains a critical bottleneck. Acoustic transducers, while widely available and commercially supported, are limited in frequency (<150 Hz), subject to amplitude variability due to preload, and produce upper harmonics that degrade wave quality [46]. Electromagnetic transducers offer improved precision but may introduce image artifacts if positioned within the FOV [90]. The GT transducer concept introduced by Runge et al. addresses many of these limitations through a constant vibration amplitude across frequencies (40–70 Hz), substantially reduced second-harmonic distortion (1.6–7.7% vs. 17.9% for acoustic), and artifact-free operation [46]. However, further clinical validation and multicenter testing of this promising technology are needed.

In addition to technical limitations, several patient-related factors modify the applicability of cardiovascular MRE. General contraindications for MRI, including non-MRI-compatible implants and claustrophobia, also apply to MRE. Iron overload disrupts gradient sequences and degrades image quality [25]. Breath-hold requirements remain challenging for cardiac patients with dyspnea, although free-breathing approaches using navigator techniques or retrospective binning are emerging [52,87].

### 11.2. Standardization and Reproducibility

A major barrier to clinical translation is the lack of standardization across cardiovascular MRE implementations. Different research groups employ widely varying excitation frequencies (24–220 Hz), pulse sequences (GRE, SE-EPI, spiral), inversion methods (LFE, DI, spherical shell, TMRE), and stiffness metrics (shear modulus in kPa vs. SWS in m/s), making cross-study comparisons extremely difficult [1,32]. The Manduca et al. ISMRM consensus provides a framework for standardized terminology and reporting, recommending that all studies report |G*| alongside the excitation frequency [32], but cardiovascular-specific consensus guidelines are lacking.

Reproducibility data are encouraging. Wassenaar et al. reported good intrasession reproducibility (concordance coefficient 0.77) and excellent intersession (0.93) reproducibility for cardiac MRE at 80 Hz [29]. Castelein et al. demonstrated excellent same-day test–retest reproducibility for cardiac multifrequency MRE with TMRE processing (ICC = 0.96 for stiffness) [63]. Dong et al. similarly reported excellent reproducibility of aortic MRE (concordance coefficients of 0.96–0.99) [87]. However, intervendor, intersite, and intersequence reproducibility studies remain to be realized.

### 11.3. Competing and Complementary Modalities

Ultrasound-based SWE has emerged as a practical and widely available technique for noninvasive assessment of cardiac stiffness. Using acoustic radiation force impulse imaging to measure myocardial stiffness, Villemain et al. were able to show a significant increase in myocardial stiffness in HCM HFpEF patients (12.68 ± 2.91 kPa) compared to a healthy volunteer cohort (4.47 ± 1.68 kPa; both *p* < 0.01) and achieved excellent diagnostic accuracy for HCM HFpEF patients (AUC = 0.993). The inherent flexibility of ultrasound-based methods, faster acquisition times, and lower costs compared to similar imaging techniques give them an advantage, especially in monitoring capabilities [9]. Natural shear-wave imaging, which exploits valve-closure-induced waves detectable by high-frame-rate echocardiography, has demonstrated feasibility in healthy volunteers and patients with cardiac amyloidosis. This suggests a possible link between the measured SWS of endogenous origin and myocardial stiffness [30,31].

Caenen et al. provided a comprehensive review of cardiac SWE approaches, noting that while ultrasound SWE offers real-time capabilities and portability, it is limited to the interventricular septum within specific echocardiographic windows and faces waveguide-related interpretation challenges similar to those of MRE [91]. These constraints reflect the broader limitations of echocardiographic techniques, which provide excellent temporal resolutions but are inherently restricted by the imaging geometry and acoustic access.

Cardiac MRE, by contrast, offers whole-heart coverage, 3D displacement encoding, and freedom from acoustic window limitations. Its acquisition is standardized, highly reproducible, and less operator-dependent. While MRE requires MRI infrastructure and longer acquisition times, it provides volumetric mechanical information that is difficult to obtain with echocardiographic techniques. With MRE and SWE addressing different clinical needs, the two modalities form a complementary toolkit rather than being in direct competition.

To contextualize MRE within the broader landscape of cardiac tissue characterization, Table 3 summarizes key attributes of commonly used modalities [9,54,92,93].

### 11.4. Artificial Intelligence and Emerging Solutions

The application of artificial intelligence (AI) and deep learning represents a rapidly developing, though still largely investigational, frontier for cardiovascular MRE. Deep learning-based approaches have been proposed as a potential alternative to conventional algorithms for MRE inversion, aiming to achieve faster, possibly more accurate stiffness reconstruction, as well as automated myocardial segmentation in MRE wave images and material parameter estimation from standard cardiac MRI. These approaches remain at an early, largely proof-of-concept stage, and their clinical role has not yet been established. Chen et al. demonstrated that a ResNet-based model could estimate myocardial material parameters from routine cine cardiac magnetic resonance (CMR) in under 1 s (compared to ~7 h for finite element analysis), achieving a <5% mean relative error in healthy subjects, although its performance degraded substantially in pathological tissue [94].

### 11.5. Future Directions

#### 11.5.1. Short-Term Achievable Developments

Several near-term developments could accelerate the clinical adoption of cardiac MRE. Multifrequency approaches such as TMRE and k-MDEV, which combine multiple excitation frequencies with advanced processing, have demonstrated improved reproducibility and spatial resolution compared to single-frequency methods [50,63,77]. Castelein et al. have emphasized that vendor-agnostic, integrated, multidriver hardware with real-time frequency modulation will be critical to replicating the clinical success of liver MRE in cardiac applications [77].

Hardware innovation is also progressing: GT transducers are a promising option for wave-generation hardware development due to their technical advantages over conventional actuation strategies, including a constant displacement amplitude across a range of frequencies and strongly suppressed upper harmonics, which could increase wave fidelity and viscoelastic reconstruction [46].

The transducer-free approach based on intrinsic cardiac motion represents an alternative pathway that eliminates hardware barriers entirely [4,55]. If combined with standardized processing, this approach could enable routine assessment of cardiac stiffness as an add-on to existing CMR protocols. However, this method currently provides only a single velocity measurement in the septum, rather than full 3D stiffness maps [4]. The transducer-free approach will benefit from future extensions, including respiratory gating to eliminate the need for repeated breath-holds, thereby enabling greater spatial coverage of elasticity estimates.

#### 11.5.2. Long-Term Research Directions

Longer-term goals include multicenter clinical trials correlating MRE-derived stiffness values with patient outcomes, the development of disease-specific stiffness thresholds for clinical decision-making, the integration of MRE with other quantitative CMR biomarkers (T1 mapping, extracellular volume fraction, strain), and treatment monitoring applications (e.g., assessing the response to tafamidis in cardiac amyloidosis) [76].

More speculative concepts include miniaturized intracardiac actuators. Morais et al. recently demonstrated myocardial shear-wave generation using a catheter-compatible transient elastography actuator in an ex vivo porcine myocardium, suggesting a potential foundation for future MRE-compatible catheter technology [56].

## 12. Conclusions

Cardiovascular MRE has evolved from initial feasibility demonstrations in phantom and animal models to quantitative disease-specific stiffness measurements in human studies, encompassing cardiac amyloidosis, HCM, diastolic dysfunction, MI, hypertension, and abdominal aortic aneurysms. Key milestones include the correlation of MRE-derived stiffness with invasive pressure–volume measurements, the development of transducer-free approaches using intrinsic cardiac motion, the introduction of multifrequency techniques with improved reproducibility, and one study suggesting that aortic MRE-derived stiffness may be associated with aneurysmal events independently of the aneurysm diameter, a finding that warrants confirmation in larger, multicenter cohorts.

Despite this substantial progress, several challenges must be addressed before cardiovascular MRE can transition from a research tool to routine clinical practice. These include the standardization of acquisition parameters and inversion methods across sites and vendors, the development of geometry-aware inversions that account for the waveguide effect in thin-walled cardiac chambers, the improvement of transducer technology for deeper wave penetration at higher frequencies, and the establishment of disease-specific stiffness thresholds through adequately powered multicenter trials. Emerging solutions, including multifrequency approaches, AI-assisted reconstruction, and free-breathing 3D acquisitions, offer promising pathways toward these goals.

The complementary development of ultrasound-based cardiac SWE further validates myocardial stiffness as a clinically relevant biomarker, and future clinical workflows may integrate both modalities alongside established CMR biomarkers (T1 mapping, extracellular volume fraction, strain) for comprehensive myocardial tissue characterization. With continued methodological standardization and validation in larger, multicenter, outcome-linked studies, cardiovascular MRE has the potential to contribute to early disease detection, differential diagnosis, treatment monitoring, and risk stratification in cardiovascular disease, although, at this stage, cardiovascular MRE remains confined to research settings, and its influence on clinical decision-making and patient outcomes has yet to be established.

## Figures and Tables

**Table 1:** Comparison of wave generation technologies for cardiovascular MRE.

Driver Type	Mechanism	Frequency Range	Advantages	Limitations	Key References
Acoustic	Pressurized air through tubing to passive drum/paddle	20–150 Hz	Widely validated and available	Frequency limited <150 Hz; amplitude varies with coupling; upper harmonics ~18%	[26,29,51,52]
Electromagnetic	Direct motor/loudspeaker driving piston/rod	20–50 Hz	Simple design; direct coupling	Bulky; MR-incompatible if too close; low frequency only	[53,54]
GT (rotational eccentric mass)	Stepper motor rotates eccentric mass via PEEK shaft	40–70 Hz	Constant amplitude across frequencies; minimal harmonics (1.6–7.7%); MR-compatible	Not yet widely available as a novel technique; limited cardiac validation	[46,49]
Driverless (intrinsic)	Aortic/mitral valve closure as natural wave source	~30–300 Hz *	No hardware; easy clinical integration; no patient discomfort	Limited to septum; variable wave amplitude; single velocity measurement; requires sinus rhythm	[4,55]
Catheter-based	Miniaturized intracardiac actuator (ultrasound)	Variable	Direct myocardial excitation; bypasses thoracic damping	Invasive; experimental only; ex vivo proof-of-concept	[56]

Abbreviations: gravitational, “GT”; magnetic resonance, “MR”; polyether ether ketone, “PEEK”. * Natural shear-wave frequency content from valve closure; not externally controlled.

**Table 2:** Pulse sequences used in cardiac MRE.

*Sequence*	*Readout*	*Gating*	*BH/FB*	*Freq. (Hz)*	*Resolution*	*Coverage*	*Advantages*	*Limitations*	*Key* *References*
*Cine GRE*	Cartesian GRE	ECG retrospective	BH (15–44 s)	60–80	2.7–5.0 mm	2D multiphase	Cardiac cycle variation; widely used	Long BH; low SNR at higher frequencies	[27,28,61,62]
*SS-SE-EPI*	SS-SE-EPI	ECG prospective	BH (~25 s)	100–140	3.0–5.0 mm	3D (five slices)	Higher SNR; shorter BH per slice	Susceptibility; ghosting; single phase	[42,50,51,63]
*rFOV* *SE-EPI*	2D selective SE-EPI	ECG prospective	BH	140	5.0 mm	3D (5 slices)	Eliminates Nyquist ghosting	Slightly rFOV	[59]
*TURBINE-EPI*	3D hybrid radial-EPI	ECG + resp. retrosp.	FB (~10 min)	100	3.0 mm iso	3D whole LV, seven phases	Free-breathing; full cardiac cycle	Long scan time; lower SNR	[52]
*Spiral GRE*	Dual-density multishot spiral	ECG prospective	BH (~23 s)	70/80/90 (MF)	2.0 mm	2D time- resolved	40 Hz temporal resolution; multifrequency	Single slice; 2D only	[50]
*Stroboscopic GRE*	Segmented spiral MRE	Pulse-triggered	BH (~25 s)	50/62.5/80 (MF)	2.1 mm	2D multislice	Multifrequency aortic; excellent ICC	Aortic only; diastolic phase only	[49]
*Pencil beam*	2D pencil beam	ECG-triggered	FB	~200–300 *	N/A (1D)	1D septum	No hardware; fast; easy integration	No map; single velocity; septum only	[4,55]

Abbreviations: one-dimensional, “1D”; two-dimensional, “2D”; three-dimensional, “3D”; breath hold, “BH”; electrocardiogram, “ECG”; echo-planar imaging, “EPI”; free-breathing, “FB”; gradient-recalled echo, “GRE”; intraclass correlation coefficient, “ICC”; isotropic, “iso”; left ventricle, “LV”; multifrequency, “MF”; magnetic resonance elastography, “MRE”; reduced field of view, “rFOV”; spin-echo echo-planar imaging, “SE-EPI”; signal to noise ratio, “SNR”; single-shot spin-echo echo-planar imaging, “SS-SE-EPI”. * Natural wave frequency. Note: Direct comparison of stiffness values across sequences and frequencies is not appropriate due to frequency-dependent viscoelastic dispersion.

**Table 3:** Comparison of cardiac tissue characterization modalities.

*Modality*	*Spatial Resolution*	*Invasiveness*	*Reproducibility*	*Availability*	*Clinical Maturity*
*Magnetic Resonance Elastography (MRE)*	Moderate (whole-heart 3D)	Noninvasive	High (standardized MRI acquisition)	Limited to MRI centers	Emerging but growing evidence base
*Ultrasound Shear-Wave Elastography (SWE)*	Localized (typically septum)	Noninvasive	Moderate (operator- and window-dependent)	Widely available	Early clinical adoption; promising monitoring tool
*T1 Mapping*	High	Noninvasive	High	Broad MRI availability	Clinically established
*Extracellular Volume (ECV)*	High	Minimally invasive (requires hematocrit)	High	Broad MRI availability	Clinically established
*Strain Imaging (Speckle-Tracking Echo)*	High (regional)	Noninvasive	Moderate (vendor- and angle-dependent)	Very widely available	Clinically established

## Data Availability

No new data were created or analyzed in this study. Data sharing is not applicable to this article.

## Supplementary Materials

The following supporting information can be downloaded at https://www.mdpi.com/article/10.3390/diagnostics16142233/s1. Table S1: Summary of human cardiac MRE and SWE studies; Table S2: Summary of aortic MRE studies.

## Abbreviations

The following abbreviations are used in this manuscript:

1D	one-dimensional
2D	two-dimensional
3D	three-dimensional
AAA	abdominal aortic aneurysm
AS	aortic stenosis
AV	aortic valve
AVC	aortic valve closure
BH	breath hold
CMR	cardiac magnetic resonance
DD	diastolic dysfunction
ECG	electrocardiogram
ECM	extracellular matrix
ED	end-diastole
EPI	echo-planar imaging
ES	end-systole
FB	free-breathing
FOV	field of view
GRE	gradient-recalled echo
GT	gravitational
Gx	imaging gradient in x-direction
Gy	imaging gradient in y-direction
Gz	imaging gradient in z-direction
HCM	hypertrophic cardiomyopathy
HFpEF	heart failure with preserved ejection fraction
HTN	hypertension
HV	healthy volunteer
ICC	intraclass correlation coefficient
IVC	isovolumetric contraction
ISMRM	International Society for Magnetic Resonance in Medicine
iso	isotropic
k-MDEV	k-space-based multidirectional elasto-viscoelasticity reconstruction
LCCC	luminal cross-sectional compliance coefficient
LFE	local frequency estimation
LGE	late gadolinium enhancement
LV	left ventricle
LVH	left ventricular hypertrophy
MEG	motion encoding gradient
MF	multifrequency
MI	myocardial infarction
MR	magnetic resonance
MRE	magnetic resonance elastography
MRI	magnetic resonance imaging
MVC	mitral valve closure
PEEK	polyether ether ketone
PWV	pulse-wave velocity
RF	radiofrequency
rFOV	reduced field of view
RR-Interval	interval between two subsequent R-waves
RV	right ventricle
S	interventricular septum
SE	spin-echo
SE-EPI	spin-echo echo-planar imaging
SNR	signal to noise ratio
SS-SE-EPI	single-shot spin-echo echo-planar imaging
SWE	shear-wave elastography
SWI	shear-wave imaging
SWS	shear-wave speed
SWV	shear-wave velocity
TE	echo time
TMRE	tomoelastography
TOF	time-of-flight
TR	repetition time
TTR	transthyretin
US-ARFI	ultrasound acoustic radiation force impulse
US-HRF	ultrasound high-repetition-frequency imaging
US-natural	ultrasound natural shear-wave imaging
US-SWI	ultrasound shear-wave imaging
US-THE	ultrasound time-harmonic elastography
