# Supplementary material for: Cardiovascular Magnetic Resonance Elastography: Current Evidence, Challenges, and Future Perspectives
Source: Diagnostics (Basel). 2026 Jul 16;16(14):2233. doi: 10.3390/diagnostics16142233 (PMC13409057; doi:10.3390/diagnostics16142233)
Supplement: Supplementary file 1 [file diagnostics-16-02233-s001.zip › Table S1.pdf]

Table S1. Summary of Human Cardiac MRE and SWE Studies

Study	Year	n	Modality	Freq (Hz)	Sequence	Inversion	Disease	Stiffness	Key Finding
Elgeti et al. [53]	2008	8 HV	MRE	24.3	GRE	Amplitude ratio	Healthy	Relative (P-V work 0.85 J)	First cardiac MRE in humans
Elgeti et al. [54]	2010	25 (10 DD, 15 HV)	MRE	24.1	GRE	Amplitude ratio	Diastolic dysfunction	Ratio 0.33 vs 0.62 (p<0.001)	First cardiac MRE in disease
Wassenaar et al. [29]	2016	29 HV	MRE	80	GRE	3D LFE	Healthy (aging)	ES 6.10±1.38, ED 4.99±1.05 kPa (p<0.0001)	Reference values; Reproducibility ICC 0.93
Arani et al. [42]	2017	8 HV	MRE	80–220	SE-EPI	3D DI	Healthy	3.8±0.6 kPa (140 Hz)	Optimal freq 140 Hz; phantom validation
Arani et al. [51]	2017	38 (22 CA, 16 HV)	MRE	140	SE-EPI	3D LFE	Amyloidosis	11.4 vs 8.2 kPa (p=0.0008)	First disease-specific cardiac MRE
Chang et al. [73]	2017	2 (1 CA, 1 HV)	MRE	140	SE-EPI	LFE	TTR amyloidosis	15.7 vs 7.2 kPa	Feasibility in TTR amyloidosis
Sui et al. [59]	2018	16 HV	MRE	140	rFOV SE-EPI	3D LFE	Healthy	7.2–7.4 kPa	rFOV eliminates ghosting
Villemain et al. [9]	2019	80 (20 HCM, 60 HV)	US-ARFI	N/A	US-SWI	SWS	HCM HFpEF	12.68 vs 4.47 kPa (p < 0.01) (AUC 0.993)	Near-perfect HCM detection
Santos et al. [31]	2019	32 (30 HV, 2 pts)	US-Natural	~30–47*	US-HFR	TOF	Healthy + CA	MVC 3.2±0.6, AVC 3.5±0.6 m/s	Natural SWI reference values
Petrescu et al. [30]	2019	63 (46 HV, 17 CA)	US-Natural	N/A*	US-HFR	Slope	Amyloidosis + aging	6.33 vs 3.54 m/s (p<0.001)	SWV increases with age and CA
Troelstra et al. [4]	2021	17 (12 HV, 5 pts)	MRE-driverless	~200–300*	Navigator	TOF	LVH, MI	14.1 vs 3.6 m/s (p=0.001)	First driverless cardiac MRE
Burnhope et al. [55]	2022	Multiple groups	MRE-driverless	N/A*	Navigator	TOF	LVH (HCM, HTN, CA)	Elevated SWV in all groups	Driverless in clinical population
Arani et al. [72]	2024	109 HV (57F, 52M)	MRE	140	SE-EPI	3D LFE	Healthy (sex/aging)	8.3±1.2 kPa; F slope 0.03 kPa/yr (p = 0.009)	Largest cohort; female-specific stiffening
Zhao et al. [74]	2024	1 HCM	MRE	N/R	N/R	N/R	HCM	21.8 vs ref 7.2–9.8 kPa	First MRE case in HCM

Meyer et al. [76]	2025	109 (54 HC, 10 LVH, 45 wTTR)	US-THE	N/A	US-THE	SWS	wTTR, LVH	3.0 vs 1.8 m/s ($p < .05$) (AUC 0.991)	Diastolic stiffness; tafamidis response
Castelein et al. [63]	2025	28 (18 HV, 10 AS)	MRE-MF	80/90/100	3D SE-EPI	TMRE	Healthy + AS (reproducibility)	SWS 2.42±0.24 m/s (ICC 0.96)	First multifreq cardiac MRE reproducibility
Anders et al. [50]	2025	11 HV	MRE-MF	70/80/90	2D spiral	k-MDEV	Healthy	ES 2.15±0.23, IVC 1.76±0.17 m/s	Time-resolved spiral MRE; ICC 0.93
Meyer et al. [52]	2026	11 HV	MRE	100	3D TURBINE-EPI	3D LFE	Healthy	3.5 (ED) – 5.7 (ES) kPa	Free-breathing 3D; seven cardiac phases

* Natural shear wave frequency content; not externally driven.

Abbreviations: two-dimensional "2D"; three-dimansional "3D"; aortic stenosis "AS"; aortic valve closure "AVC"; cardiac amyloidosis "CA"; diastolic dysfunction "DD"; direct inversion "DI"; end-diastole "ED"; end-systole "ES"; gradient-recalled echo "GRE"; hypertrophic cardiomyopathy "HCM"; heart failure with preserved ejection fraction "HFpEF"; hypertension "HTN"; healthy volunteers "HV"; intraclass correlation coefficient "ICC"; isovolumetric contraction "IVC"; k-space–based Multi-Directional Elasto-Viscoelasticity reconstruction "k-MDEV"; local frequency estimation "LFE"; left ventricular hypertrophy "LVH"; myocardial infarction "MI"; magnetic resonance elastography "MRE"; multifrequency magnetic resonance elastography "MRE-MF"; mitral valve closure "MVC"; reduced field-of-view "rFOV"; spin-echo echo-planar imaging "SE-EPI"; shear wave imaging "SWI"; shear wave speed "SWS"; shear wave velocity "SWV"; tomoelastography "TMRE"; time-of-flight "TOF"; transthyretin "TTR"; ultrasound acoustic radiation force impulse "US-ARFI"; ultrasound high-repetition-frequency imaging "US-HFR"; ultrasound natural shear wave imaging "US-natural"; ultrasound shear wave imaging "US-SWI"; ultrasound time-harmonic elastography "US-THE"; wild-type transthyretin "wTTR".

Note: Stiffness values reported in kPa (shear modulus) and m/s (shear wave speed) are not directly comparable across studies due to frequency-dependent viscoelastic dispersion and different measurement assumptions. Conversion via $\mu = \varrho \cdot c^2$ (with ϱ = 1000 kg/m^3) applies only under the assumption of a purely elastic medium
