# Supplementary material for: Cardiovascular Magnetic Resonance Elastography: Current Evidence, Challenges, and Future Perspectives
Source: Diagnostics (Basel). 2026 Jul 16;16(14):2233. doi: 10.3390/diagnostics16142233 (PMC13409057; doi:10.3390/diagnostics16142233)
Supplement: Supplementary file 1 [file diagnostics-16-02233-s001.zip › Table S2.pdf]

Table S2: Summary of Aortic MRE Studies

Study	Year	n	Design	Freq (Hz)	Sequence	Inversion	Target	Stiffness	Key Finding
Woodrum et al. [84]	2009	Ex vivo	Ex vivo	N/A	PC-MRI	Phase contrast	Porcine aorta (HTN)	Higher in HTN (histology confirmed)	Ex vivo HTN detection
Kolipaka et al. [41]	2012	8 (4 HTN, 4 NT)	In vivo	60	GRE	PG	Abdominal aorta	9.3 vs 3.7 kPa ($p=0.02$)	First in vivo HTN vs normotensive
Damughatla et al. [85]	2015	21 HV	In vivo	60	GRE	LFE	Abdominal aorta	Young 4.5, old 8.0 kPa	Stiffness + PWV increase with age
Kenyhercz et al. [86]	2016	20 HV	In vivo	60	GRE	LFE	Abdominal aorta	Higher in ES than ED ($p<0.0001$)	Cardiac cycle variation demonstrated
Kolipaka et al. [65]	2016	36 (24 AAA, 12 HV)	In vivo	60	GRE	LFE	AAA	13.97 vs 7.1 kPa ($p \leq .02$)	AAA stiffness independent of diameter
Zhang et al. [78]	2016	Phantom + 4 ex vivo	Ex vivo	60	GRE	LFE, PG	Porcine aorta	Fresh 8.4, formalin 19.3 kPa ($p=0.05$)	Regional stiffness detection validated
Dong et al. [43]	2020	31 pigs	Animal	70	Cine GRE	3D LFE	Porcine AAA	41–66% increase (all $p \leq 0.0087$)	Inverse correlation with elastin
Schaafs et al. [49]	2020	20 HV	In vivo	50/62.5 /80	Spiral GRE	k-MDEV	Thoracic + abdominal	AA 1.62, AD 2.40, AAb 2.48 m/s ($p<0.001$)	First multifreq aortic MRE reference
Dong et al. [87]	2021	40 HV	In vivo	70	SE-EPI	LFE	Abdominal aorta	~6.0 kPa; LCCC 0.96 –0.99	SE-EPI validated; excellent reproducibility
Dong et al. [88]	2022	128 (72 AAA, 56 HV)	In vivo	N/R	N/R	N/R	AAA	Lower in event group ($p<0.05$)†	Lower stiffness predicts AAA events
Mangarova et al. [89]	2022	10 mice (5 AAA, 5 ctrl)	Ex vivo	1000–1400	SE (7T)	k-MDEV	Mouse AAA	ECM-rich 1.04, ECM-poor 0.44 m/s ($p<0.05$)	Micro-MRE at 40 µm; ECM correlation $R^2=0.79$

Abbreviations: three-dimensional “3D”; ascending aorta “AA”; abdominal aortic aneurysm “AAA”; abdominal aorta “Aab”; descending thoracic aorta “AD”; control “crtl”; extracellular matrix “ECM”; end-diastole “ED”; end-systole “ES”; gradient-recalled echo “GRE”; hypertension “HTN”; healthy volunteers “HV”; k-space–based Multi-Directional Elasto-Viscoelasticity reconstruction “k-MDEV”; luminal cross-sectional compliance coefficient “LCCC”; local frequency estimation “LFE”; normotensive " NT”; phase contrast magnetic resonance imaging “PC-MRI”; phase gradient “PG”; pulse wave velocity “PWV”; spin-echo “SE”; spin-echo echo-planar imaging “SE-EPI”.
